# Coverage evaluation of mass drug administration with triple drug regimen in an evaluation unit in Nagpur district of Maharashtra, India

**DOI:** 10.1371/journal.pntd.0011588

**Published:** 2023-09-07

**Authors:** Raja Jeyapal Dinesh, Adinarayanan Srividya, Swaminathan Subramanian, Kaliannagounder Krishnamoorthy, Shanmugavelu Sabesan, Monika Charmode Raghorte, Ashwani Kumar, Purushothaman Jambulingam

**Affiliations:** 1 ICMR-Vector Control Research Centre, Puducherry, India; 2 District Malaria Officer (DMO), Nagpur, Maharashtra, India; University of Ottawa Faculty of Medicine, CANADA

## Abstract

**Background:**

Triple drug regimen (IDA; Ivermectin, Diethylcarbamazine, Albendazole) recommended for accelerating elimination of lymphatic filariasis was launched in India in December 2018. Nagpur district in Maharashtra was one of the first five districts where this strategy was introduced. The National Vector Borne Disease Control Programme (NVBDCP) at the district reported ~85.0% treatment coverage in the first round of mass drug administration (MDA) with IDA implemented in EU-2 in Nagpur district in January 2019. As per the national guideline, a coverage evaluation survey was carried out and both quantitative and qualitative data were collected to assess the treatment coverage, the level of community preparation and identify the gaps, if any, for improvement.

**Methodology:**

A Coverage Evaluation Survey (CES) following the WHO recommended protocol was conducted in one of the two evaluation units (EU-2) in Nagpur district in March 2019. Coverage Sample Builder (CSB) V2.9 tool was used to calculate the sample size, select sites and estimate drug coverage. The CSB tool followed a two-stage cluster sampling procedure to select 30 primary sampling units (ward/village as a cluster) and a list of random numbers for selecting households (HHs) in each cluster. The results were analyzed for operational indicators. Stata ver. 14.0 software was used to construct the 95% confidence limits accounting for clustering.

**Results:**

A total of 1601 individuals aged 5–85 years of both gender from 328 HHs were surveyed from the 30 randomly selected clusters in EU-2. The mean age was 33.8±17.6 years. Among the surveyed population, 78.0% received the drugs (programme reach) and 66.1% consumed the drugs (survey coverage). Survey coverage was significantly higher in rural (82.6%) than in urban (59.4%) and peri-urban (58.6%) areas (P<0.001). Directly observed treatment (DOT) among the surveyed population was 51.6%. Adverse events were reported among 6.9% respondents who reported to have consumed the drugs.

**Conclusion:**

The IDA based MDA strategy could achieve just the required level of treatment coverage (~65%) in EU-2, Nagpur district, which had previously undergone several rounds of DA-MDAs (Diethylcarbamazine, Albendazole). Having achieved an effective treatment coverage of >80% in rural areas, the coverage in urban and peri-urban areas need to be improved in order to attain the impact of IDA-MDA. It is imperative to strengthen drug delivery and community preparation activities along with improved DOT especially in urban and peri-urban areas to achieve the required level of treatment coverage. Addition of ivermectin did not have any additional perceived adverse events.

## Author summary

Government of India implemented triple drug regimen for mass drug administration (IDA-MDA) in selected districts from December 2018 to accelerate lymphatic filariasis elimination in the country. Nagpur district in state of Maharashtra, India was one of the five districts where this strategy was first implemented in January 2019 and the reported treatment coverage in one of the evaluation units in the district, EU-2 was 85.0%. This district had previously completed thirteen rounds of DA-MDA till the year 2017. TAS carried out in 2016 showed that the district did not reach elimination level, CFA < 2.0% in children aged 6–7 years. A coverage evaluation survey was carried out in EU-2 following the first round of IDA-MDA and both quantitative and qualitative data was collected to assess the treatment coverage, the level of community preparation and identify the gaps, if any, for improvement. Among the surveyed population (1601), 78.0% received the drugs (programme reach) and 66.1% consumed the drugs (survey coverage). The study showed an effective treatment coverage above 65% in rural areas and a sub-optimal coverage in urban/peri-urban areas (≤65%). There exists a challenge in achieving an effective coverage in urban and peri-urban areas with the new treatment regimen. Appropriate strategies are necessary to improve the treatment coverage in all the areas so as to achieve the elimination target in the evaluation unit. Only 51.6% DOT was observed which needs to be improved.

## Introduction

Lymphatic filariasis (LF) is targeted for elimination through mass drug administration (MDA) with two drug regimen (Diethylcarbamazine and Albendazole (DA) or Ivermectin and Albendazole (IA)). Since its inception till 2020, the Global Programme to Eliminate Lymphatic Filariasis (GPELF) has successfully delivered 8.6 billion doses to more than 925 million people at least once in 68 out of 72 endemic countries. Seventeen countries/territories have been validated as having eliminated LF as a public health problem and, five countries are currently under post-MDA surveillance [1]. However, still 863 million people in 50 countries remain at risk of LF, and require MDA [1]. To hasten global LF elimination, WHO recommended the use of triple drug regimen (Ivermectin, Diethylcarbamazine and Albendazole; IDA) for MDA (a) for implementation units (IUs) that have not started or have fewer than four effective rounds of DA, (b) for IUs that have not met the appropriate epidemiological targets in sentinel and spot-check site surveys or in TAS despite meeting drug coverage targets; and (c) for communities where post-MDA or post-validation surveillance identified infection suggesting local transmission [2]. With LF elimination target year of 2020 bygone, the WHO has set 2030 as the new target year for its elimination, aligned with the sustainable development goals [1,3,4].

India contributes 40% to the global LF burden from 328 endemic districts spread over 21 States and Union Territories [5–8]. The national LF elimination programme with DA-MDA launched in 2004 covered all the endemic districts in India. By the beginning of 2021, 174 districts were under MDA and 98 had stopped MDA and are going through various stages of transmission assessment surveys (TAS) that shows whether the prevalence of infection in the evaluation unit (EU) has been lowered to a level below which transmission is no longer sustainable and recrudescence is unlikely to occur even in the absence of MDA [5–8]. As a part of the accelerated LF elimination plan (2018), Government of India introduced IDA-MDA in five districts initially in 2019; and gradually up scaled to 36 more districts by 2022 [5–9].

**Fig 1:**
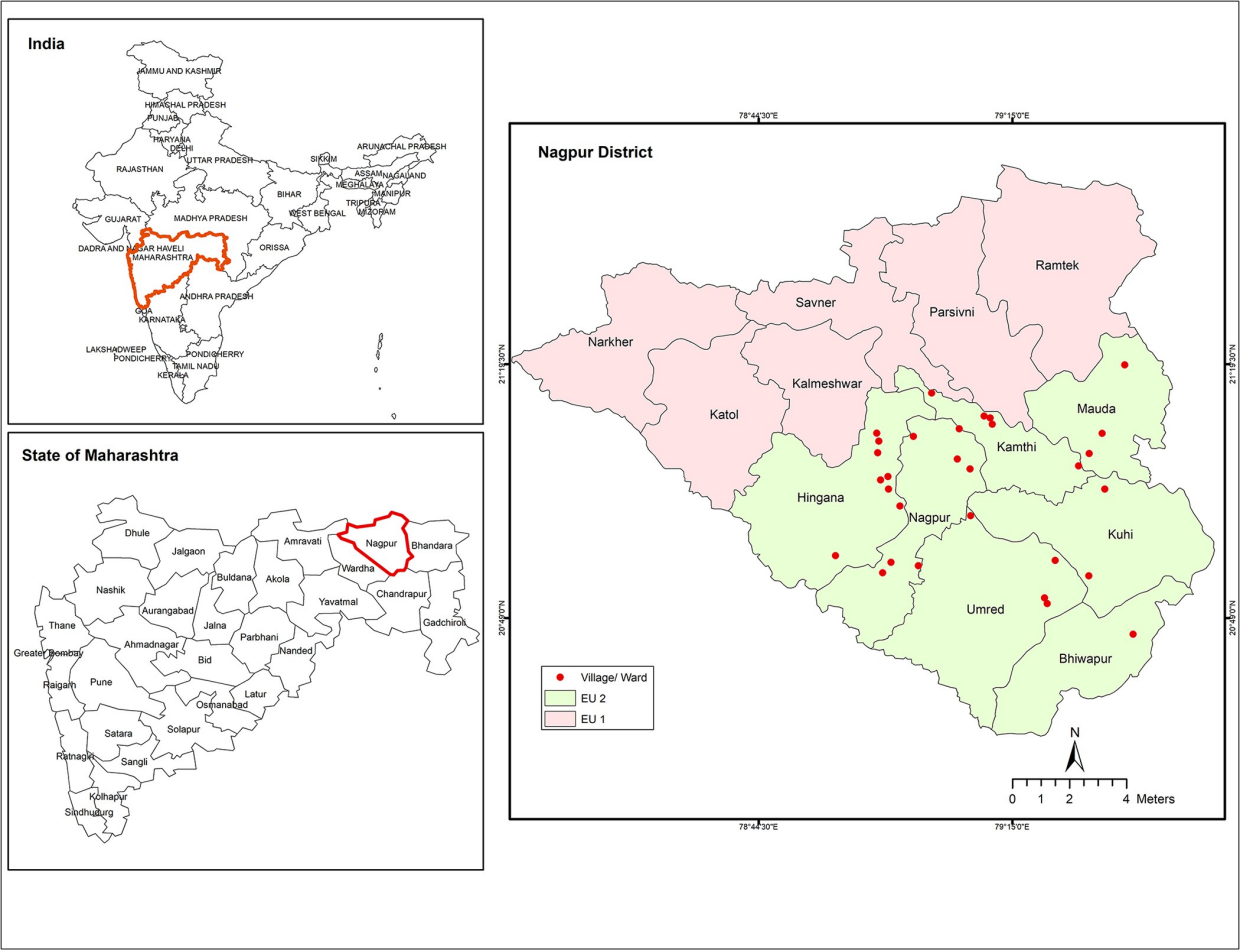
Map showing the evaluation units and study sites in Nagpur district, Maharashtra, India. EU-1 Evaluation Unit 1; EU-2 Evaluation Unit 2. Base layer of map: http://www.diva-gis.org/Data. License information: https://www.diva-gis.org/docs/DIVA-GIS_manual_7.pdf. ArcGIS Software: https://www.arcgis.com.

Nagpur was one of the five districts chosen for implementation of IDA-MDA as both EUs namely Nagpur North (EU-1) & Nagpur South (EU-2) in the district failed in TAS-1 despite 13 DA-MDA rounds since 2004 (Fig 1). MDA with IDA was implemented in the entire district in January 2019. In order to estimate the treatment coverage and assess the perception of the community towards the new triple drug regimen and its delivery, an independent coverage evaluation survey (CES) was carried out in 2019 following the MDA in Nagpur EU-2. The district NVBDCP had reported a treatment coverage of ~85.0% in EU-2 based on the data reported by all drug administrators with denominator estimated from existing population enumeration records available with the district health department.

## Material and Methods

### Ethics statement

The study was approved by the Institutional Human Ethics Committee (IHEC-0118/D). The purpose of the survey was explained clearly to the head of the family or the eldest member in the family and a blanket informed written consent was obtained for the family prior to the interview. All methods were carried out in accordance with relevant guidelines and regulations in the Declaration of Helsinki.

### Study setting

**Fig 2:**
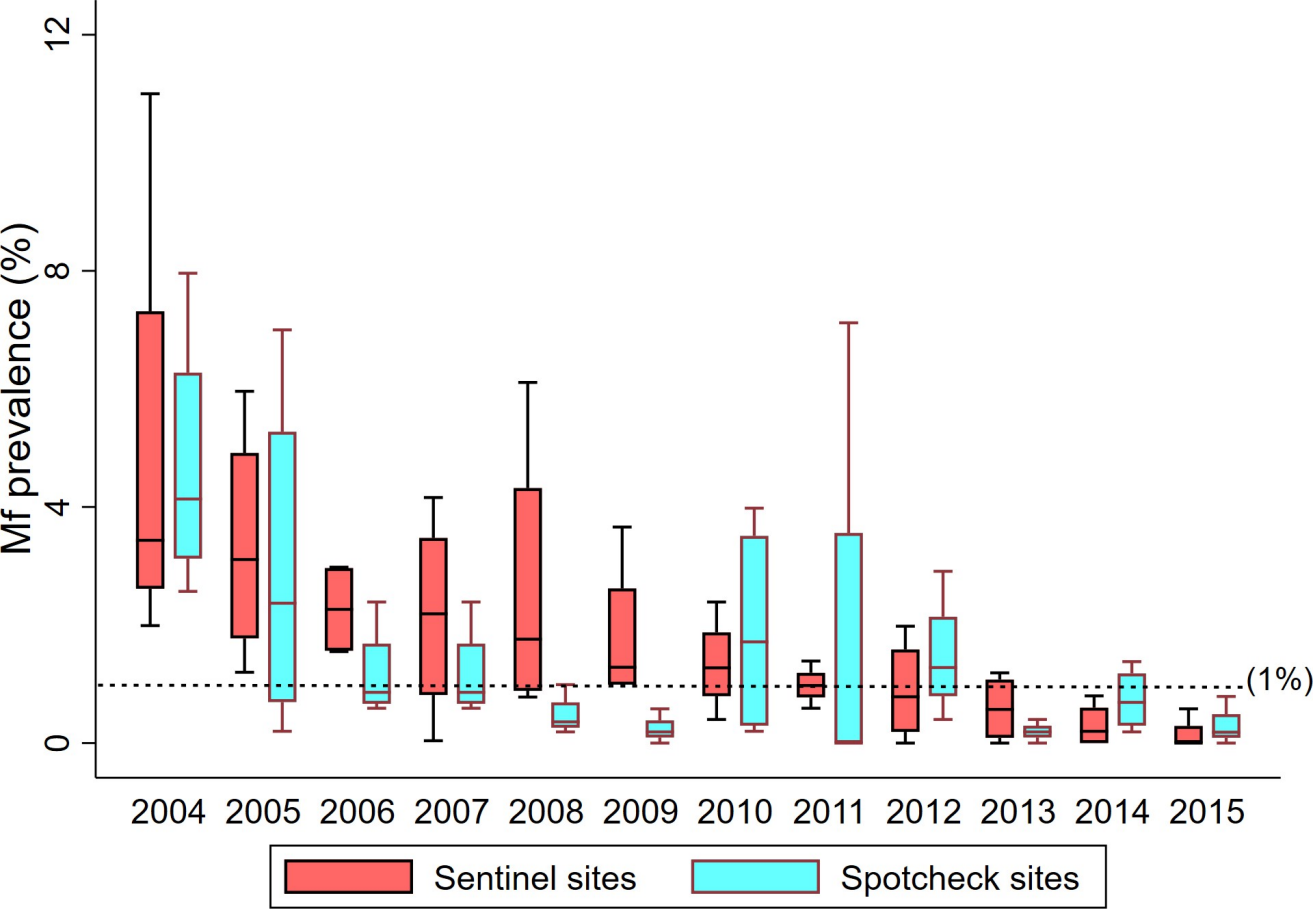
Box plot of Mf prevalence in sentinel and spot-check sites in Nagpur (2004–2015) [5]. —**—:** WHO threshold Mf prevalence.

Nagpur district in the state of Maharashtra, India with an estimated population of 5.1 million is spread over an area of 9892 km^2^ and is divided into 14 sub-districts consisting of 1900 villages and 176 wards [10]. Thirteen rounds of DA-MDA have been completed till the year 2017 excluding 2016 (DA-MDA was not carried out in 2016 due to TAS). Coverage reported by the district programme was above 89.0% in all the rounds, ranging from 89.0 to 96.3% in different years (2004–2017). Independent assessment of DA-MDA coverage during the same period ranged between 58.0% and 87.0% with suboptimal coverage (<65.0%) reported during the years 2012 (63.0%) & 2015 (58.0%) [5]. The reason for low coverage in the year 2015 was attributed to fear in the community due to a death that occurred due to aspiration of drug, which had a serious impact on MDA as informed by district programme. However, the microfilaria (Mf) surveys conducted in spot-check (3 rural & 1 urban) and sentinel sites (3 rural & 1 urban) since 2004 showed significant decline from 4.97% to <1% in 2015 (Fig 2) [5]. Survey in 10 additional sites also showed a low level of Mf rate (0.15%) [5]. As Mf rate was observed to be <1% in all spot-check and sentinel sites including 10 additional sites in 2015, the district qualified for TAS. The district was divided into two EUs (excluding Nagpur Municipal Corporation), each with population less than ῀2 million for the purpose of TAS and a school-based TAS was conducted in both EUs in 2016. The TAS results showed that the number of 6-7-year-old children positive for circulating filarial antigen (CFA) was above the critical cut-off (Number of CFA positive children ≤ 20) for passing TAS in both the EUs (EU-1: 40 children were CFA positive out of 1931 screened, EU-2: 38 children were CFA positive out of 1289 screened. Hence, DA-MDA was continued in 2017.

First round of IDA-MDA in Nagpur: IDA-MDA was introduced in 2019 to accelerate LF elimination in the district. The MDA commenced on 20^th^ January 2019 in the entire district and continued until 29^th^ January 2019 in rural areas and till 5^th^ February 2019 in urban areas considering the large population to be covered. This was immediately followed by mop-up rounds to cover all the left out beneficiaries, particularly in anganwadis, schools, colleges, government and private offices besides other areas. (Anganwadi centre is a government establishment in the village that takes care of the nutrition, provide health screening and health education to children aged 0–6 years, adolescent girls, pregnant and lactating mothers. The workers at the village level who deliver the services are called Anganwadi workers. They act as the community’s primary link with the health services and all other services.) Extended mop-up rounds were organized in densely populated urban and peri-urban areas that consistently reported low coverage in previous DA-MDA rounds. Special outreach camps were also organized in industrial areas, brick kilns, factories, transport hubs such as bus-stands, and railway stations to cover the left out individuals. The mop-up was carried out till 10^th^ February 2019.

### Community drug administrators (CDAs)

Accredited Social Health Activists (ASHAs), Anganwadi Workers (AWWs), Auxiliary Nurse Midwife (ANMs), and Multipurpose Workers (MPWs) who are part of district health system acting as an interface between the community and the public health system, were engaged as CDAs by the district health department. The CDAs were trained to assess the eligibility for IDA-MDA (children <5 years, pregnant/lactating mothers and seriously ill were ineligible), calculate dosage based on age (for DEC) and height (using dose pole for IVM) and to provide treatment to eligible individuals by house-house visits and in schools. Additionally, student volunteers from nursing and pharmacy colleges were trained and engaged as CDAs to cover the densely populated urban and peri-urban areas. Around ~2000 CDAs were engaged for drug administration in EU-2 alone with a target of 150–200 HHs each.

An incentive of INR 750/- was given to each CDA for administration of drugs to 50 HHs. The CDAs were supervised by health assistants, health supervisors, technicians, lab scientific officers and medical officers. One supervisory staff was allocated for supervising every 5–9 CDAs. The supervisory staff were trained (in batches) regarding the IDA programme over a period of 8 days and the CDAs were trained (in batches) over a period of 6 days (14-19^th^ January 2019) prior to the roll out of MDA.

IDA dosage: A single oral dose consisting of diethylcarbamazine (age-based dose as per national guidelines: 2–5 years- 1 tablet (100mg); 6–14 years- 2 tablets of 100 mg each; and ≥15 years 3 tablets of 100 mg each), albendazole (400mg fixed dose) and ivermectin (height-based as per WHO guidelines using colour-coded dose poles: 90–119 cm- 1 tablet (3 mg); 120–140 cm- 2 tablets of 3 mg each; 141–158 cm 3 tablets of 3 mg each; and >158 cm 4 tablets of 3mg each) was given to all eligible individuals. Children aged 2–5 years were administered diethylcarbamazine (1 tablet of 100 mg) and albendazole (400 mg fixed dose) as per the programme guidelines.

### Drug administration

Drugs were administered through booths established in schools, anganwadi centers and health facilities as well as by door-to door approach. Mobile booths were established at transit hubs, brick kilns, industries, factories etc., during the mop-up rounds. Stenciling on the walls/doors of houses administered and finger marking of individuals who received drugs were practiced for ease of identification. Special rapid response teams headed by a Medical officer were deployed (with vehicle) for attending to adverse events following drug administration at Primary Health Centre (PHC) level.

### Community preparation & IEC activities

Community preparation was carried out through print/electronic media and social media few weeks prior to roll-out of MDA with the support of stakeholders following the strategies recommended in the national guideline [5]. Awareness rallies, wall paintings/writings, school awareness programs and sensitization of public representatives, faith-based organizations, community and media were carried out extensively prior to the launch of IDA-MDA program as described elsewhere [8]. IEC materials such as banners, billboards, brochures, posters, handbills, newspaper advertisements etc., and social mobilization activities were supported by Project Concern International (PCI), Delhi. Advocacy, communication and media sensitization was carried out by Global Health Strategies (GHS). A popular actor & youth icon was made brand ambassador to popularize the importance of addressing LF as a public health problem and acceptance of IDA by the community [8].

### Study design & period

A community based 30 cluster cross-sectional survey was carried out between 26^th^ March 2019 – 30^th^ March 2019 in EU-2 (Nagpur South). This evaluation unit having a population of 1,412,012 covering seven sub-districts was selected for the survey as it accounted for more number of infected individuals in the TAS (prevalence of CFA among 6–7 years olds in EU-1- 2.1% & EU-2- 2.9%).

### Sample size

Coverage Survey Builder (CSB) v2.9 tool, recommended by WHO was used to select the sites, estimate the sample size and obtain the frequency of household selection [11]. The sample size (900) was based on the assumed coverage of 65% at 95% confidence level, absolute precision of 6.5%, with design effect of 4 and a non-response rate of 10%.

### Sampling method

Details of all villages/wards (cluster) with population and estimated number of households in EU-2 were obtained from the State Health department. The CSB tool used a two-stage cluster sampling procedure for the selection of clusters and households (HHs) within each selected cluster. In stage I, 30 clusters were selected with probability proportionate to the estimated population size in each cluster. In stage II, HHs were selected systematically in proportion to the total number of HHs in the selected cluster with a minimum of 5 HHs in each cluster. For this purpose, the tool rolled out two lists (A and B) of random numbers of which one (namely list B) was randomly selected and used for all study sites to ensure uniformity among the survey teams. By this process, a total of 900 individuals were expected to be interviewed to estimate coverage accuracy within ±6.5%.

### Data collection method

Three teams each with two technical staff trained in sociological survey and one local guide/interpreter familiar with the study area and local language (Marathi) were involved in the data collection. The survey was carried out in the morning and evening hours when most of the people were available at home. Upon arrival in the selected study site, each team walked through a predetermined route, enumerating from the first house as the initial house. The household corresponding to the number on the selected HH list was chosen and information regarding participation in MDA was collected from all members aged ≥5 years in the HH. Parents/caregivers provided information regarding drug consumption for children aged 5–12 years. The houses which were found locked at the first visit were not revisited, rather the survey was continued with the next selected HH following the sampling frequency based on the proportion to the total number of HHs in all the selected sites. With a minimum of five households in each site (as per the CSB output), the number of HHs visited was based on the total number of HHs in the given site. Responses from those who were physically present at the time of the survey were only included, and proxy responses for the absentees were not collected.

The CES questionnaire consisted of two parts - the first part was for obtaining information from all the available individuals in the selected households on drug consumption, reasons for non-consumption, adverse events etc. The second part collected additional information regarding awareness and attitude towards LF & MDA programme, source of information on MDA, participation in previous MDA rounds, and willingness to participate in future rounds. This information was obtained from the head of the family or one of the available adult members in each HH surveyed using an interview guide. Besides, the challenges during the roll-out of IDA-MDA were discussed during a review meeting held with the district health officials such as Assistant Director of Health services (ADHS), District Malaria Officer (DMO), Medical Officers and health supervisors during the conduct of the CES. Discussions were also held with drug administrators such as ASHAs, and anganwadi workers in the study sites.

### Statistical analysis

Demographic details were expressed as percentages. Coverage indices (programme reach, surveyed coverage with their 95% confidence interval and compliance) were obtained from the CSB v2.9 tool and the results were interpreted as per the guidelines mentioned in the tool [11]. Coverage was further analyzed area-wise i.e, urban, peri-urban and rural areas as per Census 2011 criteria [10]. Comparison of coverage between the gender was carried out using χ2 and Fisher’s exact test, whichever applicable. Logistic regression analysis was carried out to compare the difference in programme reach and surveyed coverage between genders after adjusting for cluster random effect in the model. A P-value of <0.05 was considered significant. The statistical software Stata ver. 14.0 was used to construct the 95% confidence limits accounting for clustering [12].

Key terms:

- Target population: Individuals who are eligible to receive the drugs, based on the criteria for drug safety, and is usually 85–90% of the total population. Eligible are individuals aged 5 years and above excluding pregnant/lactating mothers and seriously ill [11].
- Reported coverage: It is the number of people who were reported by all drug administrators as having ingested drugs divided by the total population in the area [11].
- Survey coverage (SC): Coverage estimated through the use of population-based survey sampling methods. It is calculated as a proportion. The denominator is the total number of individuals surveyed and the numerator is the total number of individuals surveyed who were identified as having ingested the drug [11].
- Programme reach coverage (PC): The proportion of the people in the survey area who received the preventive chemotherapy, regardless of whether the drug was ingested or not [11].
- Compliance: The proportion of people in the survey area who received and also swallowed the drugs during MDA [11].

## Results

**Fig 3:**
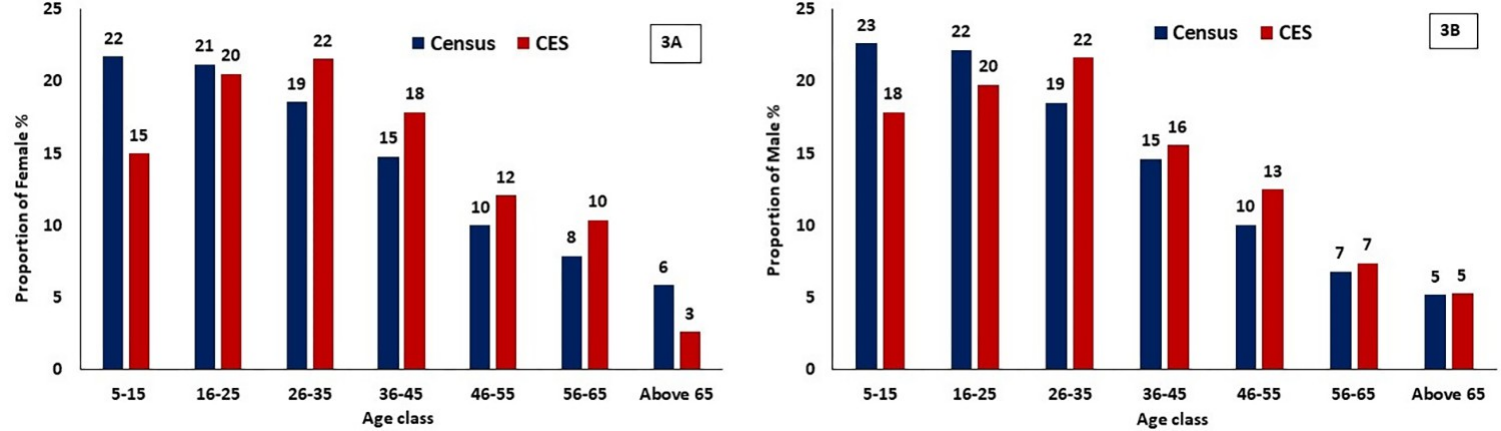
Comparison of age-distribution of samples in the coverage evaluation survey (CES) with (3A) Census population- Female; (3B) Census population- Male.

A total of 1601 individuals aged 5–85 years from 328 HHs were surveyed from 30 selected clusters following the aforementioned sampling method. The average family size was 5 (Range 2–19). Of the total surveyed, 840 (52.5%) were males and the remaining 761 (47.5%) were females (F:M ratio 0.9:1). The age and gender distribution of the study population was almost comparable with Census population [10] (Fig 3A and 3B). The mean age was 33.8±17.6 years. Among those surveyed, 1558 (97.3%) individuals were found to be eligible for drug administration and the remaining 43 (2.7%) were ineligible as they were either pregnant or lactating mothers (0.9%), or sick (1.7%) at the time of drug administration.

### MDA coverage

**Fig 4:**
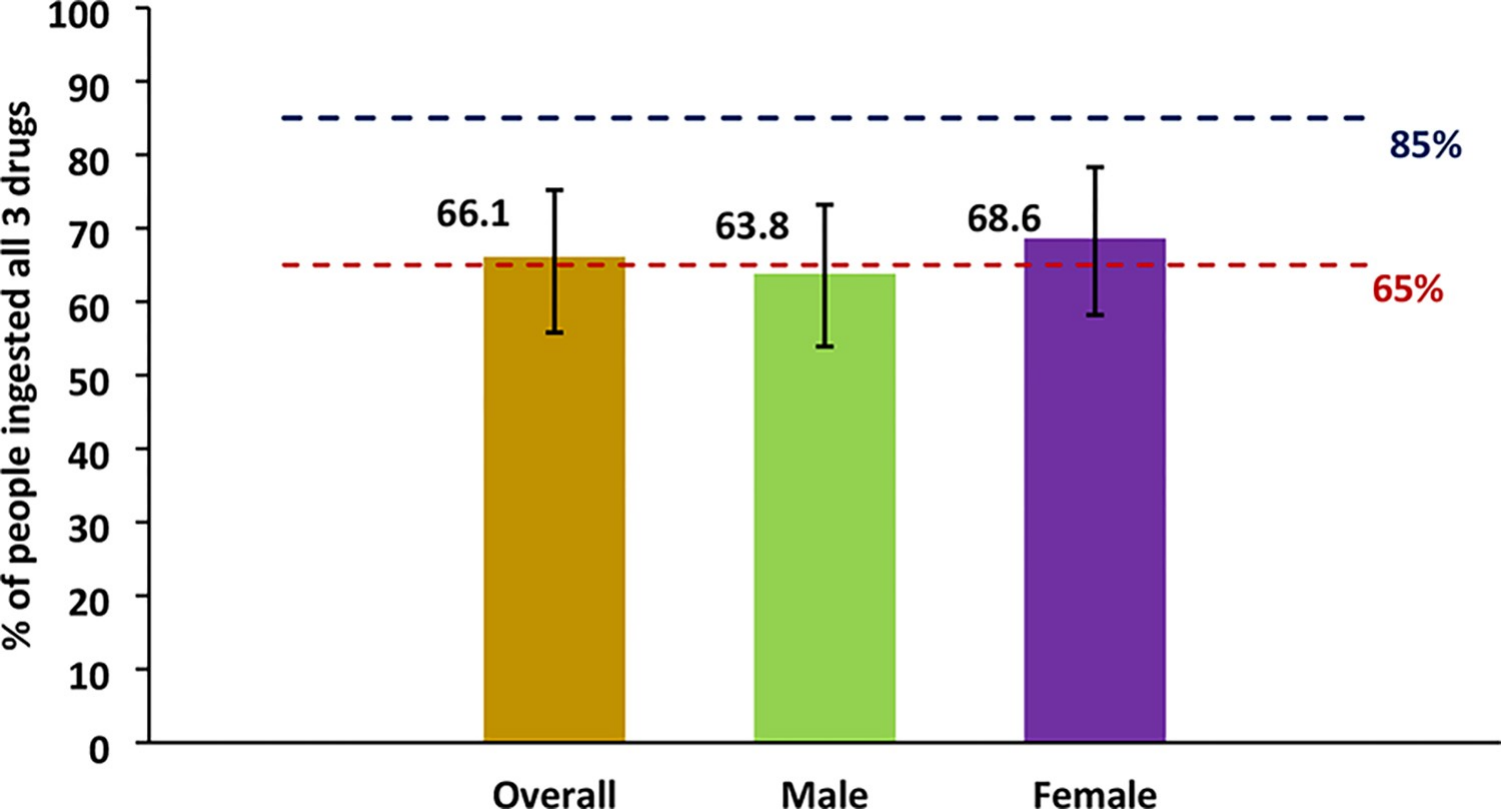
Estimated Survey coverage by gender in EU-2, Nagpur. Blue dash: Reported coverage. Red dash: WHO target coverage threshold.

Of the 1601 surveyed population, 1249 reported to have received all the three drugs with an overall programme reach coverage of 78.0% (95% CI: 68.3–85.4). It was 79.3% among females and 76.5% among males, the difference was not statistically significant (P = 0.14). The survey coverage was 66.1% (95% CI: 55.6–75.2) and was more among females (68.6%) compared to males (63.8%). The gender difference in survey coverage was statistically significant (P = 0.043) (Fig 4).

Table 1 compares the survey coverage across rural, urban and peri-urban areas in EU-2. The sample distribution was approximately similar to the census population across areas. Comparison of the survey coverage showed that it was significantly higher in rural compared to the urban and peri-urban areas (P < 0.001).

**Table 1:** Area-wise surveyed coverage in EU-2, Nagpur.

Areas	#clusters	#Population (% of total)	#Surveyed (% of total)	# received	Programme reach coverage%	#consumed	Survey coverage%
Rural	15	28889 (23.7)	484 (30.2)	452	93.4	400	82.6
Urban	6	36257 (29.8)	458 (28.6)	323	70.5	272	59.4
Peri- urban	9	56616 (46.5)	659 (41.2)	474	71.9	386	58.6
Overall	30	121762 (100)	1601 (100)	1249	78.0	1058	66.1

### Compliance

Of 1249 who received the drugs, 1058 (84.7%) reported to have consumed the drugs. The overall non-compliance rate (proportion individuals who received but did not consume the drugs) was 15.3%. It was 16.6% (95%CI: 14.0–19.7) among males and 13.9% (95% CI: 11.3–16.8) among females. Self-reported supervised consumption (DOT) among the surveyed population was 51.6%.

### Reasons for non-receipt / non-consumption of drugs

Among the surveyed (n = 1601), 500 (31.2%) individuals did not participate in MDA due to various reasons. Of these 500, 176 (35.2%) did not receive the drugs due to non-delivery, 112 (22.4%) refused to receive the drugs, 169 (33.8%) received but did not consume and 43 (8.6%) received but did not consume all the tablets due to many number of pills (Table 2). Among those who did not participate in IDA-MDA (n = 500), 181 individuals (36.2%) were of the opinion that it was not necessary to take drugs in the absence of any disease/symptoms. Also, they had no trust in the quality of drugs that were administered in loose form without expiry date. Another 65 (13.0%) refused or did not consume due to fear of adverse events.

**Table 2:** Reasons for non-receipt and non-consumption of drugs (N = 500).

Non-participation in MDA	Reason	n (% of n)			
		All areas	Urban	Peri- urban	Rural
Drugs not received due to issues with drug delivery (N = 176) (35.2%)	Aware of MDA but drugs not delivered by CDA	89 (17.8)	18 (20.2)	61 (68.5)	10 (11.3)
	Unaware of MDA & drug not delivered by CDA	25 (5.0)	0 (0.0)	25 (100)	0 (0.0)
	Out of station	62 (12.4)	37 (59.7)	20 (32.3)	5 (8.0)
Refused to receive drugs (N = 112) (22.4%)	Not having the disease or at risk of infection and drugs are not required	88 (17.6)	35 (39.8)	40 (45.5)	13 (14.8)
	Fear of adverse events	24 (4.8)	19 (79.2)	5 (20.8)	0 (0.0)
Drug received but not consumed (N = 169) (33.8%)	Not interested in taking drugs due to low risk perception	90 (18.0)	21 (23.3)	45 (50.0)	24 (26.7)
	No trust in quality of drugs	3 (0.6)	0 (0.0)	0 (0.0)	3 (100)
	Fear of adverse events	41 (8.2)	15 (36.6)	15 (36.6)	11 (26.8)
	On treatment for other illnesses	16 (3.2)	4 (25.0)	8 (50.0)	4 (25.0)
	Away from home at the time of CDA visit*	19 (3.8)	11 (57.9)	5 (26.3)	3 (15.8)
Drugs partially consumed (N = 43) (8.6%)	Many pills to swallow	43 (8.6)	0 (0.0)	36 (83.7)	7 (16.3)
Total		500 (100)	160 (32.0)	260 (52.0)	80 (16.0)

*Family members received the drugs from CDA

### Self-perceived adverse events

Adverse events were reported by 73 (6.9%) individuals amongst 1058 who reported to have consumed the drug. This was more common among females (67.1%) than males (32.9%). Some of the commonly reported adverse events were giddiness/dizziness (54.8%) followed by nausea (17.8%), vomiting (15.1%), headache (12.3%) and fever (8.2%). Palpitation & shivering (4.1%), fatigue (2.7%) and diarrhoea/abdominal pain (2.7%) were rarely reported. These were self-limiting or were managed by the local health staff with ORS/antipyretics/anti-emetics, etc.

### Additional information from the survey

As mentioned in the Methodology, additional information regarding awareness and attitude towards LF & MDA programme, source of information on MDA, participation in previous MDA rounds, and willingness to participate in future rounds was obtained from the head of the family or any available adult member in each of the surveyed HHs. As many as 328 individuals were interviewed and their mean age was 42.6±12.9 years (range 18–75 years), of whom 180 (54.9%) were females and remaining males.

A majority (63.1%) of the respondents had some knowledge about LF transmission, it’s cause and clinical features of the disease while 35.4% were not aware of LF at all. Misconception on LF transmission was observed among 1.5% of the respondents. This included exposure to polluted water/air, God’s curse and some sort of imbalance in the body. The major source of information regarding MDA was from a health worker or a CDA (78.1%), followed by friend, neighbour or relative (2.4%), electronic media (2.1%), and pamphlets/posters (1.2%). About 53 (16.2%) individuals were not aware of IDA-MDA, of these majority (75.5%) were from urban/peri-urban areas and remaining were from rural areas. The most liked aspects of MDA were treatment by house visits (49.0%), administration of drugs at no cost (29.4%), knowledgeable CDAs (21.1%) and riddance of head louse infestation (0.5%). The reasons cited for the disinclination to MDA were fear of adverse events (42.6%), lack of follow-up for adverse event management during previous MDA rounds (42.6%) and time of visit by CDA being inconvenient for people (14.8%). About 10.6% of the respondents felt the number of tablets should be reduced to improve compliance. With regard to participation in previous MDA rounds, 161 (49.1%) had participated in 1–4 rounds, 57 (17.4%) in 5 or more rounds while 110 (33.5%) had never participated in the previous MDA. Among those never participated (n = 110) previously, 59 (18.0%) did not participate in the current round of IDA-MDA also. These comprised the ‘never treated’ population that included 52.5% males and rest females. Most (81.4%) were in the economically productive age group 24–60 years and rest were above 60 years. Also 41 (69.5%) and 13 (22.0%) were from the peri-urban and urban areas respectively. Majority 295 (89.9%) were willing to participate in future rounds of MDA while the rest were either unwilling or uncertain about their participation.

### Observation on community perspective

In urban and peri-urban areas the CDA had visited during the afternoon hours, when most working people were not available at home. In addition, student volunteers were involved in drug administration in these areas owing to the dense population. People were reluctant to consume drugs in these areas due to lack of trust and a common notion as to ‘*Why consume drugs distributed by youngsters*!’. Some of these volunteers were reported to have distributed drugs without adequate explanation of its purpose to the community and were in a hurry to complete their targets. To ensure supervised consumption, CDAs did not administer the drug to individuals who refused to take them at the time of visit, citing reasons like ‘*I will have drugs later after having food’*/ ‘*I will have after completing daily chores’* etc. During the earlier DA-MDA rounds, the practice of leaving the drugs for the absentees with other available family member was followed. However, during IDA-MDA the CDAs were instructed to treat the eligible population only by DOT, not leaving the drug for the absentees. Those individuals who were missed during the previous visit were treated during the subsequent mop-up rounds if available. Lack of perceived need for consumption of drugs was also reported. A 62-year-old male said *“I’m old*, *I need no drugs at this age*”. Yet another conveyed her family members would consume the drugs only if advised by their family physician.

In most rural areas, the drugs were administered by the CDAs in the morning and late evening hours when most members would be available at home. In the evening, the people were administered drugs post dinner and were advised to sleep. This ensured people from experiencing mild side effects post drug administration such as dizziness, giddiness, drowsiness, fatigue etc., go unnoticed. In few villages, motivated CDAs went to the extent of visiting agricultural fields during lunch (it was time of the year when chilies were harvested), where the people work during the day to ensure drugs were administered. Additionally, the medical officer from the area health centre or equivalent official assisted the drug administration teams to the field which helped in developing confidence and encouraged public to participate in the MDA. Besides, people informed that regular announcement about IDA-MDA was made through microphones in the entire villages using vehicles, during panchayat meetings and also during religious meetings. Graffiti’s and slogans informing the day of IDA-MDA were also observed on the walls of village houses by the survey team.

### Providers perspective of challenges in urban/ peri-urban areas

- Lack of effective and adequate IEC activities: The community preparation activities were carried out by a pre-identified stakeholder just a few days before the MDA using available IEC materials. The role of district health system was limited to co-ordination of field level IEC activities. There was no mechanism to monitor and evaluate the impact of these IEC activities.
- Shortage of drug administrators & overburdened CDAs: Due to inadequate number of drug administrators, the target given for each CDA was three times higher (~150 HHs) than the recommended level (50HHs). Additionally, the field level health workers were involved in other health related activities in some areas.
- Certain areas experiencing a high refusal rate & non-compliance during previous MDA rounds continued to remain a challenge.
- Non-availability of certain group of people such as migrants, and daily wagers despite repeated visits.

## Discussion

Clinical trials with a single dose of IDA have shown over 90.0% of microfilaricidal effect was sustained for 5 years [13]. IDA-MDA required less number of rounds with an effective coverage of 65% when compared to DA-MDA [14]. While clinical trials are carried out under controlled conditions, there may be several confounding factors that may affect treatment coverage when administered under community settings. A multi-centric community trial revealed that IDA had comparable safety and acceptability profile with DA [15–17]. Following the recommendation of WHO, Government of India started implementing IDA-MDA initially in five selected districts in December 2018 with a plan to upscale in phases to accelerate LF elimination in the country [18]. A CES was carried out in one of the EUs of Nagpur district (EU-2) that underwent its first round of IDA-MDA in early 2019 and the reported coverage in EU-2 was 85.0%.

Though the overall survey coverage was 66.1%, the lower limit of 95% confidence interval (55.7%) was much lower than the WHO target threshold for LF elimination indicating a sub-optimal coverage in many clusters. The low treatment coverage was mainly in urban/ peri-urban areas suggesting the need for strengthening community preparation activities prior to MDA and intensify DOT in these areas. According to the CES protocol, the survey coverage should be within 10.0% of reported coverage, however it was found 18.9% lower than the reported coverage (85.0%) suggesting a likely problem with the reporting system [11]. Use of latest information technology to capture data on real time basis will help in avoiding errors in reporting, if any, as well as aid in documentation for future use. The programme reach coverage (78.0%) in the study area was higher than the survey coverage suggesting that a proportion of people did not ingest the drugs upon its receipt indicating the need for further strengthening supervised drug administration. To cater to the large population in urban/peri-urban areas, IDA-MDA round was planned for more number of days compared to rural areas and also were supported by additional manpower (student volunteers) for drug administration. Based on concurrent reports from supervisors, extended mop-up rounds and special out-reach camps to treat the left-out individuals in urban and peri-urban areas were organized by the programme. With these amplified efforts, a programme reach coverage of only ~70.0% could be achieved in urban/peri-urban areas as most working class people were not available at the time of drug administration. We observed that a high proportion of individuals in urban and peri-urban areas were either away from home or out of station at the time of drug administration. Novel strategies adapting to the life style of people in the urban/peri-urban areas may be required. The surveyed coverage estimates were below 60.0% implying the drugs were not consumed by all the people who had received it. This stresses the need for strictly ensuring DOT in urban/peri-urban areas. A survey conducted among 1096 IDA-MDA eligible participants from 240 households (40 clusters x 6 HH) in selected urban slum areas catered by one urban primary health centre in Nagpur city in the month of February 2019, reported a much lower programme reach coverage (55.2%), survey coverage (48.5%) and supervised consumption (28.9%) [19]. Survey coverage as low as 50.3% and 23.0% has been reported in previous rounds of DA-MDA especially in urban and slum areas of Maharashtra (Nagpur and Solapur district) and a similar trend was observed in this study in urban areas [20,21]. Involvement of young student volunteers as CDAs to meet manpower shortage was not well received by the community in urban/peri-urban areas. Similar observation was reported by a study in Sri Lanka [22]. Also, the time of drug administration (afternoon hours in urban areas) may have influenced the coverage estimates in urban/peri-urban areas. Additionally, in an attempt to ensure supervised consumption, CDAs did not administer drugs to individuals who did not comply, and did not revisit them owing to their work commitments. The programme reach coverage was ~70.0% in urban and peri-urban areas much lower when compared to rural areas (93.4%). Nearly 22.0% individuals of which majority were from peri-urban and urban areas reported that the drugs were not delivered by the CDA. As this situation is related to drug delivery by the health system, appropriate measures are needed to ensure adequate number of trained drug administrators are deployed besides strengthening the community preparation of the urban population for compliance. It is also necessary to train the drug administrators on good interpersonal communication skills which will encourage people to participate in the MDA round.

Contrastingly, the rural areas documented a good coverage (PC- 93.4% & SC- 82.6%) much higher than the required coverage threshold. Strategies such as administration of drugs in the late evening hours’ post-dinner, ensured people did not experience much of the side effects commonly associated with consumption of the drugs as they retire in bed for the day. A low proportion of individuals in rural areas refused to receive drugs compared to urban and peri-urban areas. Also, fear of adverse drug events was reported less from rural areas. Personal involvement of medical officers in the field during drug administration brought confidence among the people and encouraged their participation in MDA corroborates with findings of available literature [22].

About 18.0% individuals were ‘never treated’ (i.e., either did not receive the drugs or received but did not consume the drugs) in any of the rounds of MDA including the first round of IDA-MDA. Majority of them were in the economically productive age group of 24–60 years from urban/peri-urban areas. As the country moves towards elimination, it is imperative to understand the reasons for such non-participation by certain portion of people. Strategies should be developed to fast-track the identification, encourage and treat such non-compliant individuals. With weak reliability of the responses to participation in previous rounds (as many as 13 rounds), one should be cautious in interpreting the never treated population data. Fear of adverse events was reported by 13.0% individuals more commonly in urban and peri-urban areas. Efforts should be made to allay fears about the drugs and also promote additional benefits associated with IDA treatment such as riddance of head louse, and deworming that will be a potent motivational tool to consume the drugs. A strong community-specific IEC campaign and mop-up activity following a feedback from supervisors’ coverage tool will help to improve the community acceptance and ensure required level of coverage in every supervisor’s area with about 500 HHs [11]. Adverse events were reported among less than 10.0% participants that were mostly self-limiting and was comparable to the observations from previous studies [16,19]. Fortunately, no severe adverse events were reported in the study area.

Analysis of additional information gathered revealed that 35.4% respondents were not aware of LF while 1.5% had misconceptions. This suggests the need for intensified community awareness efforts prior to drug administration as indicated by other earlier studies in the State [19–22]. Among 53 individuals who were not aware of IDA-MDA, majority (75.0%) were from urban/peri-urban areas where low treatment coverage was observed. This suggests the need for enhanced community preparation activities prior to MDA. In view of the dense population in urban/peri-urban areas and mobility of the population, appropriate drug delivery strategies specific to communities with different profile of occupation and migration need to be adopted to enhance coverage. It was observed that the major source of information regarding MDA was from a health worker or a CDA (78.1%) and house visit was the most preferred approach for MDA. These results indicate the importance of interpersonal communication not only during MDA but also during the house visits for any other preventive care. In addition, adequate training and orientation of all drug administrators with focus on communication and inter-personal skills must be ensured as it will encourage better community participation. Non-consumption or partial consumption of drugs due to many number of pills was expressed as a concern by the respondents which is a universal challenge that may be addressed by strict implementation of supervised consumption. The other reasons for non-receipt/non-consumption of drugs corroborated with findings from other such studies [19–23].

Available literature reveals that several studies reported coverage estimates (on DA) using different methodologies making it difficult to compare the estimates [19–23]. The present study is one of the few studies which assessed coverage using the WHO-CSB tool following inclusion of ivermectin in MDA in India [19–24]. One of the limitations was that the responses were self-reported and depend on the recall memory of participants. Though this survey was conducted within 2 months of IDA-MDA, a few parents of children were confused with other health program that involved mass administration of drugs such as deworming for soil transmitted helminths (STH) infection conducted as a part of National Deworming Day held on 10^th^ February and 10^th^ August every year. The surveyed coverage is based on the total number of individuals in the surveyed houses including children of all age classes. In the absence of data on the actual family size, the estimated surveyed coverage based on the surveyed sample of 5 years and above (considering their eligibility for IDA-MDA) may be an overestimate in this study. The houses found locked at the first visit were not revisited owing to time constraints, rather was continued with the next selected HH from the generated list of random numbers. Replacing locked HHs by available HHs might have resulted in overestimation of coverage. This study did not capture the socio-economic background of individuals that may have influenced their participation in MDA as discussed in other studies [22]. The area-wise Mf prevalence could be another factor that influences the treatment coverage. However, such disaggregated data for the study areas is not available for analysis. Also, the data from a small sample from urban area may influence the overall coverage level. In the perspective of programmatic assessment, the coverage estimates are applicable for the implementation unit as a whole irrespective of rural and urban population which varies across districts. The sample size calculated was for the entire EU-2, hence, the results on area-wise coverage should be interpreted with caution as the study was not powered enough for making such area-wise comparisons. More studies on treatment coverage with IDA-MDA from different settings may be useful to identify the potential gaps which when addressed will enhance treatment coverage.

## Conclusion

Coverage evaluation survey of first round of IDA-MDA showed the surveyed treatment coverage was optimal at the threshold level ~65.0% (66.1%; 95%CI 55.7–75.2) in EU-2 of Nagpur district. Having achieved an effective treatment coverage of >80% in rural areas, the coverage in urban and peri-urban areas need to be improved in order to attain the desired impact. To improve coverage in these areas more concrete efforts towards community preparation/sensitization, a strong IEC and innovative strategies to capture the mobile/ working population are required. The Government of India has recently unveiled a renewed five-pronged strategy for accelerated elimination of LF by year 2027, one of which is conducting mission mode MDA with dates synchronizing with the National Deworming Day. Ten endemic States have been identified to implement MDA in February and the remaining in August 2023 [25]. With the introduction of current block level evaluation strategy [25], there is a scope to segregate high risk areas (blocks with urban population) for targeted approach. The study identified 18.0% individuals as never treated in the community. As we move towards LF elimination it is imperative to device strategies to encourage their participation in future MDA rounds.
